# Use of a Novel Theory-Based Pragmatic Tool to Evaluate the Quality of Instructor-Led Exercise Videos to Promote Youth Physical Activity at Home: Preliminary Findings

**DOI:** 10.3390/ijerph20166561

**Published:** 2023-08-11

**Authors:** Lexie R. Beemer, Wendy Tackett, Anna Schwartz, Melia Schliebe, Alison Miller, Andria B. Eisman, Leah E. Robinson, Thomas Templin, Susan H. Brown, Rebecca E. Hasson

**Affiliations:** 1 School of Kinesiology, University of Michigan-Ann Arbor, Ann Arbor, MI 48109, USA; abeemer@umich.edu (L.R.B.); anschwa@umich.edu (A.S.); mhsch@umich.edu (M.S.); alimill@umich.edu (A.M.); lerobin@umich.edu (L.E.R.); ttemplin@umich.edu (T.T.); shcb@umich.edu (S.H.B.); 2 iEVAL, Battle Creek, MI 49015, USA; wendy@ieval.net; 3 College of Education, Wayne State University, Detroit, MI 48202, USA; aeisman@wayne.edu

**Keywords:** cognitive load, instructor-led exercise videos, pediatric health, pediatric intervention

## Abstract

Background: Exercise videos that work to minimize cognitive load (the amount of information that working memory can hold at one time) are hypothesized to be more engaging, leading to increased PA participation. Purpose: To use a theory-based pragmatic tool to evaluate the cognitive load of instructor-led exercise videos associated with the Interrupting Prolonged Sitting with ACTivity (InPACT) program. Methods: Exercise videos were created by physical education teachers and fitness professionals. An evaluation rubric was created to identify elements each video must contain to reduce cognitive load, which included three domains with four components each [technical (visual quality, audio quality, matching modality, signaling), content (instructional objective, met objective, call-to-action, bias), and instructional (learner engagement, content organization, segmenting, weeding)]. Each category was scored on a 3-point scale from 0 (absent) to 2 (proficient). A video scoring 20–24 points induced low cognitive load, 13–19 points induced moderate cognitive load, and less than 13 points induced high cognitive load. Three reviewers independently evaluated the videos and then agreed on scores and feedback. Results: All 132 videos were evaluated. Mean video total score was 20.1 ± 0.7 points out of 24. Eighty-five percent of videos were rated low cognitive load, 15% were rated moderate cognitive load, and 0% were rated high cognitive load. The following components scored the highest: audio quality and matching modality. The following components scored the lowest: signaling and call-to-action. Conclusions: Understanding the use of a pragmatic tool is a first step in the evaluation of InPACT at Home exercise videos. Our preliminary findings suggest that the InPACT at Home videos had low cognitive load. If future research confirms our findings, using a more rigorous study design, then developing a collection of instructor-led exercise videos that induce low cognitive load may help to enhance youth physical activity participation in the home environment.

## 1. Introduction

The current generation of children are the first to have their entire childhood influenced by the internet and mobile devices such as smartphones and tablets [1]. This trend was exacerbated during the COVID-19 pandemic when screen time and internet use were ubiquitous as academic learning transitioned to online platforms. This public health crisis also created an opportunity to develop and promote innovative solutions utilizing technology, such as online video platforms, to encourage physical activity engagement in young people. Promoting physical activity during childhood is a national priority, as less than 25% of children and adolescents aged 6–17 years meet the physical activity guidelines in the United States [2]. With online learning and many daily activities conducted via the internet, it is an opportune time to utilize virtual exercise programs to promote youth physical activity at home.

Currently, there are several opportunities for children to view and participate in online physical activity videos. Exergames (i.e., video games that incorporate movement to progress in the game) have shown promise to enhance physical health by using technology to encourage physical activity participation. For example, a study by Silva et al. found that exergaming provided the same acute physiological benefits as conventional exercise in a group of young adults [3]. Exergames have also been shown to help increase physical activity participation in children. A meta-analysis found that exergames induced similar physiological and psychological responses compared to laboratory-based exercise in children [4]. Teachers have also used exercise videos in elementary school classrooms to encourage movement throughout the day [5,6,7]. Just Dance is one popular resource freely available in video format via YouTube where participants mimic players’ dance moves for the duration of a popular song. Another resource is GoNoodle which is an online program targeted at families and educators and provides a variety of interactive movement breaks to use in the classroom or home [8].

Another online resource, the Interrupting Prolonged Sitting with Activity (InPACT) at Home program is an evidence-informed physical activity program that utilizes instructor-led exercise videos [7]. The InPACT at Home program was developed during the COVID-19 pandemic to address the limited access to structured physical activity opportunities for young people due to shelter-in-place restrictions [7]. This program was rapidly adapted from the InPACT classroom-based physical activity intervention currently being implemented in elementary schools across the state of Michigan [6]. For the InPACT at Home program, physical education teachers, fitness professionals, pediatric exercise physiologists, and athletes from across the state of Michigan were hired to develop exercise videos that were developmentally appropriate and could be completed at home with no or minimal equipment. The InPACT at Home program is one of few home-based physical activity programs that utilize virtual methods to encourage young people to be active at home. This program is freely available (http://inpactathome.umich.edu, accessed on 7 August 2023) and broadcast on public television (https://michiganlearning.org/show/inpact-at-home/, accessed on 7 August 2023). However, the quality of these online resources has not been established as there is no validated tool to evaluate the quality of exercise videos designed to increase youth physical activity engagement.

**Figure 1:**
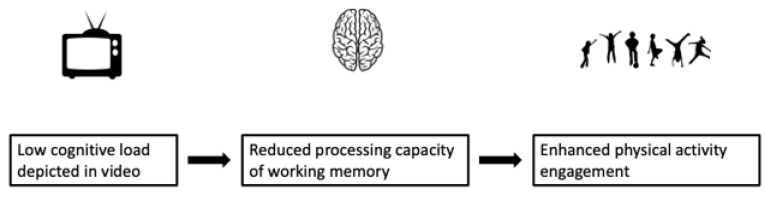
Video quality conceptual model.

One way to determine the quality of exercise videos is to consider the amount of cognitive load they induce, which is a key determinant of viewer engagement. The cognitive load theory has been extensively used to guide the creation and evaluation of educational videos. This theory builds on the model of human information processing to reduce the demands on the viewers working memory to help them engage effectively [9,10,11]. The processing capacity of working memory is limited in the amount of information that can be comprehended at one time; therefore, the cognitive load theory can be used to guide the design of verbal and visual instructions that are given to the viewer [12,13] (See Figure 1). For example, exercise videos that will encourage physical activity engagement include components such as presenting the instructions for each exercise clearly and in an organized format, providing a clear demonstration of each activity, reinforcing correct form and giving encouragement throughout the activity [14].

Further, there are three main types of cognitive load that an instructor needs to consider when creating online exercise videos to reduce the demands on working memory to improve engagement: (1) the level of difficulty of instructions presented (intrinsic load), (2) the mental energy needed to link current knowledge to the new information (germane load), and (3) the distractions that prevent processing new information (extraneous load) [15,16]. When creating exercise videos, the developer/instructor must give attention to these three types of cognitive load. This ensures that the video does not exceed the viewer’s processing capacity—and increases their ability to follow the instructions successfully. In summary, instructors should work to manage intrinsic load, optimize germane load, and minimize extraneous load.

The cognitive load theory has not been used to assess the quality of structured exercise videos; however, it has been used to examine motor skill acquisition. For example, Rekik et al. assessed gender differences in learning baseball skills from video modeling vs. static pictures [17]. Authors also assessed the mental effort participants invested during both the video modeling and static picture conditions using a validated self-report questionnaire to measure cognitive load. A 9-point Likert scale ranging from (1) very, very low mental effort to (9) very, very high mental effort was used. This study found that females reported lower mental effort during the video modeling condition compared to the static pictures condition, while there were no differences found in males. Additionally, females reported significantly higher mental effort during the static pictures condition compared to males, with no gender differences observed in the video modeling condition.

Previous research in the education literature has also demonstrated that instructional videos that induce a low cognitive load (i.e., higher quality) are associated with increased participant learning and performance. In a study that used cognitive load theory principles to create video-based and live instructor-led online training sessions and their effects on performance outcomes, authors found that participants performed significantly better on easy tasks compared to more difficult ones independently of the type of instruction used [18]. Further, Donker et al., found that higher-quality videos, determined by learner satisfaction and acceptance, included text depicted on the screen, images, and clear sound (i.e., components associated with lower cognitive load) which improved learner motivation, interest, and skills to complete the assignment [19].

Cognitive load has primarily been taken into consideration in previous studies that utilize video-based methods to improve youth learning outcomes. However, there are no pragmatic tools available that enable fitness professionals and physical education teachers to evaluate the quality of exercise videos. This is important, as many general and physical education teachers are using exercise videos to promote youth physical activity in their classrooms and gyms [20].

The overarching objective of this study was to use a theory-based pragmatic tool to evaluate the cognitive load of the InPACT at Home instructor-led exercise videos. More specifically, we examined differences in cognitive load by video type (i.e., cardio, strength, mindfulness, and sports skills). This study is a first step toward the construction and validation of a publicly available tool that can be used to determine the cognitive load of instructor-led exercise videos. Data generated from this study will provide a systematic process to evaluate instructor-led exercise videos. From this evaluation, future researchers can use a more rigorous study design to assess video quality. Our long-term goal is to be able to develop and disseminate a pragmatic tool that enables us to generate video improvement recommendations to enhance existing videos and provide guidance for developing new instructor-led exercise videos that promote youth physical activity engagement.

## 2. Methods

### 2.1. Exercise Videos

There were a total of 132, 8-min exercise videos created by 14 instructors. The InPACT at Home exercise videos are organized into the following categories: (1) cardio-based (i.e., videos incorporate circuit training with the goal of reaching at least a moderate intensity with example movements such as jumping jacks) (2) strength-based (i.e., videos include muscle-strengthening activities by incorporating movements such as push-ups), (3) sports skills (i.e., videos teach motor skills to support lifelong movement such as throwing a ball), and (4) mindfulness (i.e., videos promote calmness with the incorporation of yoga poses and breathing exercises).

Examples of equipment used were basketballs, a dining room table chair, weights, or heavy objects (e.g., water jug, soup can), a tennis ball, steps on a staircase, tape, a jump rope, a towel, a shirt, a wall, and a soccer ball. The exercise videos can be found on the InPACT at Home website (http://inpactathome.umich.edu, accessed on 7 August 2023). The current study was observational and did not evaluate the opinions of children. However, youth mood, attitudes, and recommendations for improvement after engaging in InPACT at Home exercise videos were previously evaluated in a pilot study [21]. This study found that overall, young people reported positive mood responses after participating in the videos. In addition, young people were satisfied with the video structure, enjoyed having an opportunity to move at home, and reported liking the engaging instructors. Suggestions for improvement were also given. These findings have been submitted for publication. The current study utilized quantitative content analysis to analyze pre-recorded, instructor-led exercise videos from the InPACT at Home program. This study was approved by the University’s Institutional Review Board (HUM00192745).

### 2.2. Exercise Video Evaluation Rubric

An external evaluation team (composed of the expert program evaluator (W.T) and two additional evaluators from iEval services) was previously contracted to develop the video evaluation rubric. These evaluators each have over 15 years of extensive experience using observational rubrics in early childhood, K-12, after-school programs, and university instructional settings. This rubric was created to help with the development of the InPACT at Home program. Table 1 displays the rubric used to independently evaluate all 132 InPACT at Home exercise videos. The rubric was developed based on recommendations derived from the tenants of the cognitive load theory to identify key elements that each video must contain to reduce extraneous cognitive load, optimize germane cognitive load, and manage intrinsic cognitive load [14,22]. To develop the rubric, the evaluation team looked for a validated instrument that specifically addressed video quality but was unable to locate one. Accordingly, the evaluation team reviewed the existing peer-reviewed literature related to determining video and instructional quality and created the rubric based on those findings [14,22]. The external evaluation team then pilot-tested the tool with a small cohort of the videos. For the pilot test, each of the three reviewers evaluated the videos independently using the rubric guidelines created, then came together and held a norming discussion (i.e., a process in which a team meets to establish and agree upon a set of guidelines) to determine the final rubric categories and scoring criteria.

The rubric consisted of three domains which included four components each. The first domain is the technical domain. The technical domain is defined as the aspects of the video that relate to its visual and audio communication to the viewer. Within the technical domain the components are *visual quality* (i.e., the video is visually clear), *audio quality* (i.e., the audio is clearly understood and heard), *matching modality* (i.e., the visual/pictorial and audio/verbal content are communicating the same information), and *signaling* (i.e., on-screen text or symbols are used to highlight important information). For example, in a video leading to low cognitive load for the technical domain, the viewer can clearly see and hear what the instructor is saying and doing. The instructor demonstrates the movements as they are being explained verbally, and on-screen text such as what the sets and reps for the movements are included.

The second domain is the content domain. The content domain is defined as the instructor’s stated objectives of the video and the instructor’s ability to meet those objectives. Within the content domain, the components are *instructional objective* (i.e., objective is stated explicitly), *met objective* (i.e., the stated objective is fully met in the video), *call-to-action* (i.e., an explicit call to continue being physically active for the learner is communicated), and *bias* (i.e., the content is free of gender, racial, and religious biases). An example of a video leading to low cognitive load for the content domain would be a video in which the instructor clearly states the goals and how the participant will reach those goals, works to meet those goals throughout the video, ends the video with encouraging next steps to continue being active, and is free of bias.

The third domain is the instructional domain. The instructional domain is defined as how the tasks and directions given by the instructor in the video are organized. Within the instructional domain, the components are *learner engagement* (i.e., instructor encourages learner participation), *content organization* (i.e., content is presented in an organized, logical way), *segmenting* (i.e., information is presented in short sequences), and *weeding* (i.e., information that does not contribute to the learning goal or help build relationships is eliminated). An example of a video leading to low cognitive load in the instructional domain is one in which the instructor continuously encourages the learner, presents the information in organized short segments and deletes any information that does not support the goal.

### 2.3. Video Evaluation Training

The internal evaluation team consisted of three research staff (L.R.B, A.S., and M.S.) members who were trained by a member of the external evaluation team (W.T.) during a 2-h virtual training session to understand the rubric elements and use the rubric to evaluate the exercise videos. Prior to the training session, the research staff reviewed the rubric. During the training session, the external evaluator verbally described the domains and components in the rubric and gave relevant examples of how each component would appear in the video. Once the rubric was understood, the internal evaluation team watched two videos and independently evaluated them for practice. After each video was evaluated, scores were discussed as a team with the external evaluator, and discrepancies were identified. Where discrepancies were present the internal evaluation team discussed why they chose their scores and ultimately came to a consensus. This allowed the internal evaluation team to become familiar with the rubric in practice and to have the opportunity to ask the external evaluator clarifying questions before starting data analysis.

### 2.4. Video Evaluation Plan

Data analysis of the InPACT at Home videos occurred from September 2021 to March 2022. The three video evaluators independently scored each of the videos. The evaluator assessed all videos from one instructor to better identify common areas of improvement based on instructor style. For example, if one instructor created 10 videos, all 10 videos were evaluated before evaluating another instructor. After the evaluators completed an independent review of approximately 20–30 videos, the team met to discuss their independent evaluations for each video. If there were differences for any of the components between the three evaluators, then the team discussed that score and came to a consensus. When clarifying questions occurred during the evaluation process, the video evaluation team reached out to consult the program evaluator. This process occurred until each of the three individuals evaluated all 132 videos. The symmetry of the evaluator’s performance was measured using an intra-class correlation coefficient and the interrater reliability after the independent evaluation and prior to consensus was moderate at 0.72.

### 2.5. Video Scoring and Data Analysis

For each component within the three domains, the evaluation team used the rubric to determine the points that should be awarded based on whether the key component was absent, developing, or proficient (see detailed descriptions of scoring criteria in Table 1). A score of 0 points indicated that the key component was absent, meaning it did not exist in the video. A score of 1 point indicated that the key component was developing, meaning it existed in the video but there was room for improvement. A score of 2 points indicated that the key component was proficient, meaning it existed in the video and did not need to be improved. An example of a score of 0 in the technical component of visual quality would be that the video was blurry for the entire duration, making it difficult for the viewer to see the instructor moving. An example of a score of 1 would be that the video was blurry for parts of the video. Additionally, an example of a score of 2 would be that the video was visually clear during its entire duration. Videos with a high score (more components rated as proficient) displayed low cognitive load and videos with a low score (fewer components rated as proficient) displayed high cognitive load.

A sum score of all 12 components within all three domains was the total score for each video. Videos could score up to a total of 24 points. Videos with total scores below 13 points were classified as having high cognitive load, scores between 13–19 points were classified as moderate cognitive load, and scores between 20–24 points were classified as low cognitive load. The mean total overall video score was calculated by adding the individual composite scores of all videos and dividing by the total number of videos. The mode for each individual category was also calculated and the components in least and most need of improvement were highlighted to inform video improvement. A Shapiro–Wilk test was performed to test the normality of the data and showed that the distribution of total cognitive load scores departed from normality (*p* < 0.01). We subsequently used a non-parametric Kruskal–Wallis H test to assess differences in overall video scores by video type. Data were analyzed using SPSS Version 27.

## 3. Results

A total of 132 exercise videos were evaluated from September 2021–March 2022. The mean total score for the videos was 20.1 ± 0.7 points out of 24. The mean total score for the cardio videos was 20.0 ± 0.7. The mean total score for the mindfulness videos was 20.2 ± 0.9. The mean total score for the sports skills videos was 20.2 ± 0.5. The mean total score for the strength videos was 20.3 ± 0.6. There were no significant differences in overall video score by video type (*p* > 0.05). 

**Figure 2:**
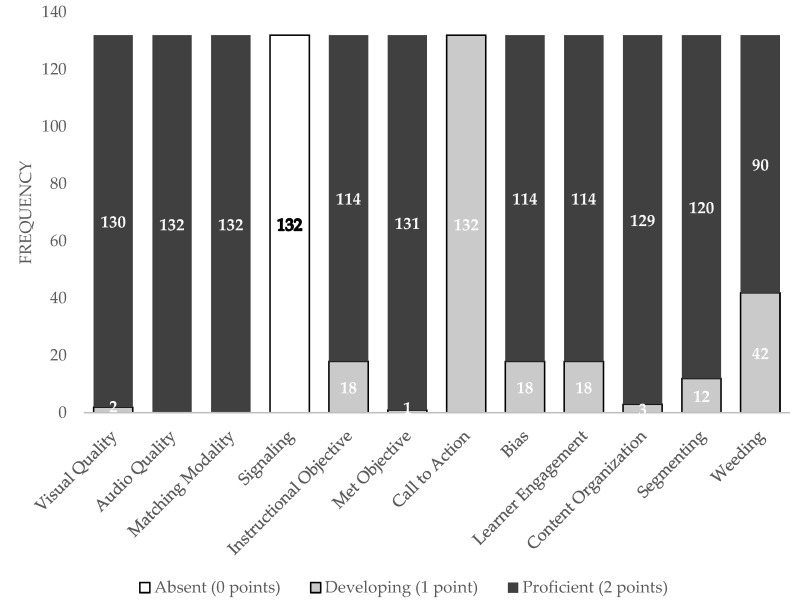
Frequency of scores by video category.

In terms of inducing cognitive load, there were 112 videos (85%) classified as low (scoring between 20–24 points), 20 videos (15%) classified as moderate (scoring between 13–19 points), and 0 videos (0%) classified as high cognitive load (scoring less than 13 points). Figure 2 illustrates the frequency of scores across the 12 components within the three domains. All domains scored a 2 out of 2 except signaling (technical domain; mode of 0 out of 2) and call-to-action (content domain; mode of 1 out of 2).

## 4. Discussion

The overarching objective of the current study was to use a theory-based pragmatic tool to evaluate the cognitive load of instructor-led exercise videos associated with the InPACT at Home program (http://inpactathome.umich.edu, accessed on 7 August 2023). Given the widespread dissemination of these exercise videos during the COVID-19 pandemic, there was a need to evaluate the exercise videos for quality; however, no validated assessments were readily available to do so [7]. Accordingly, an evaluation rubric was created to evaluate the cognitive load to understand if the InPACT at Home exercise videos exceeded the viewer’s processing capacity and if so, to determine the area(s) most in need of improvement. Our analysis determined that we have preliminary evidence suggesting that the overwhelming majority of InPACT at Home videos are high quality as 85% of videos induced low cognitive load, 15% induced moderate cognitive load, and 0% induced high cognitive load. The videos scored the highest in the instructional domain, followed by the content and then technical domains. The components in the most need of improvement was *signaling* (technical domain) and *call-to-action* (content domain). This study is the first step in developing a systematic process by which to evaluate exercise video content.

Previous research has shown that video quality is associated with viewer engagement. For example, a meta-analysis found that videos with on-screen instructors significantly increased student motivation to engage in the lesson by providing social cues (i.e., face and social gestures) in the videos [23]. However, Mayer demonstrated that instructor presence can increase cognitive load and distract the learner from the content [24]. Therefore, when instructors are present it is important to focus on the visual, audio, and organizational components to reduce the overall cognitive load. Castro-Alonso et al., conducted a review of the five strategies of the cognitive load theory that have been shown to optimize instructional materials [11]. The suggested strategies instructors should try to include are the following: (1) include both text and visualizations, (2) integrate the text and visualizations, (3) only include key information, (4) use signaling to point out key information, and (5) segment the information by adding pauses between different instructions. Similarly, Zheng et al. found that segmenting videos into smaller parts of information resulted in lower levels of cognitive load and assisted a student’s learning [25]. The length of online instructional videos has also been shown to affect student engagement. Guo et al., found that students’ median engagement time was approximately 6 min at most [26]. Additionally, Afify et al., found that instructional videos shorter than this were more effective in increasing student achievement and retention of information and had decreased cognitive load compared to longer videos [27]. These findings suggest that the quality of instructor-led videos, specifically instructor social cues and the duration of the videos, are important to student-level engagement and learning outcomes and should be taken into consideration when designing virtual-based programs.

While the InPACT at Home videos were developed with components to decrease cognitive load, instructors were given creative agency for what to include within the given duration of the videos. In the current study, within the three domains, the videos scored the lowest in the technical domain (mode: 6.0 out of 8). The technical domain is important because it assesses the ability to clearly see, hear, and understand what is being asked of the learner throughout the video and when performed well can help increase retention and transfer of information as well as increase engagement [26,28].

In the current study, the component in the technical domain that was most in need of improvement was signaling—as it was absent from all videos. Signaling is defined as on-screen text or symbols that are used to highlight important information throughout the video. Signaling has been shown to improve a student’s ability to retain and transfer new knowledge [28,29]. In a study assessing the addition of signaling to educational videos to improve vocabulary in preschoolers, it was found that children performed better on vocabulary tests after visual signaling cues (i.e., outlining the words on the screen by circling them when talked about and inserting arrows to point where the child’s attention should be) were added to the video [30]. Accordingly, InPACT at Home instructors can incorporate the use of a timer so that students have a clear understanding of the duration of each exercise. Second, verbally expressing the number of repetitions or time that will be spent in each exercise can be a helpful addition in combination with providing this information as text onscreen. These suggestions will help to improve understanding so that the viewer can focus their energy on exercising and not on figuring out what is being asked of them.

The InPACT at Home videos scored the second highest in the content domain (mode: 7 out of 8). The content domain is important because it assesses if there is a clear objective and material in the video as well as ensures that the video works to meet the stated objective throughout. Assessing content helps to analyze if the video is accurate, useful, and free from bias. In the content domain, the component most in need of improvement for the InPACT at Home videos was call-to-action. Call-to-action in this study is defined as the instructor making a clear and direct statement encouraging the viewer to continue to be physically active each day. A reason that call-to-action scored low could be because each of the videos used the same script for the ending and only reminded the child to fill out a daily exercise log to keep track of their activity without including a specific encouraging call to stay active after using the video. A recommendation to improve the call-to-action in the videos would be to explicitly tell the viewer to keep using the videos each day to help them reach the recommended 60 min of physical activity needed to maintain health. Another recommendation to improve the content domain score of videos is to ensure that every video is self-contained and does not refer to a previous or next video since students may not be watching them in that order. Referring to the other videos would distract the learner from the current video objectives leading to a higher cognitive load.

Finally, InPACT at Home videos scored high across all four components in the instructional domain (mode: 8 out of 8). The instructional domain is important because it assesses how the information is included and organized in the video. The lowest component score in the instructional domain for the InPACT at Home videos was weeding. The weeding component refers to eliminating information that does not contribute to the learning goal or help build relationships between the instructor and viewer. Weeding, or the deletion of extraneous information, has been shown to improve retention and transfer of knowledge and lower learning difficulties [31]. Brame recommended the following to improve the weeding component of videos: (1) eliminate loud music that may overpower the instructor’s voice, making it difficult to understand their directions, (2) eliminate complex backgrounds that may visually distract the viewer away from the instructor who is demonstrating the movements, and (3) eliminate words and movements that are not developmentally appropriate for the viewer [12]. For example, some instructors in the InPACT videos used words such as “sagittal plane” and “transverse abdominals” when describing activities. It is recommended to use accurate language; however, the instructor should define those words so that they do not negatively impact cognitive load. Overall, understanding the domains and components most in need of improvement can guide the development of new instructor-led physical activity videos.

A few strengths of this study should be noted. First, the rubric was developed by an experienced program evaluator and is grounded in theory. Second, the videos were analyzed by three reviewers which minimized potential data analysis errors and allowed for higher reliability and trustworthiness of the analysis. Third, the videos were professionally filmed in a controlled studio environment. Fourth, the videos were designed and performed by certified physical education teachers and fitness professionals. The present study also had limitations. First, the video review rubric has not yet been validated. Future studies should assess the psychometric properties of the rubric to determine its validity. Second, the videos were professionally developed and recorded in a public broadcasting studio, thereby limiting their generalizability to other recording environments. This is important to mention, as many physical education teachers and fitness professionals are recording amateur exercise videos using tablets and cellphones in their homes and gyms [32]. Future research should use a more robust experimental design to determine the validity of the video evaluation rubric in various recording environments.

## 5. Conclusions

The current study used a pragmatic tool informed by theory to evaluate the cognitive load of the InPACT at Home instructor-led exercise videos. Understanding the cognitive load induced by instructor-led exercise videos is a novel area of research and has important implications for the utility of such a resource to promote sustained physical activity participation in young people. This was the first step in creating a systematic process to evaluate the quality of exercise videos. An important next step is to employ a more robust experimental design to assess the psychometric properties of our video evaluation rubric to determine its validity. Once established, future video developers (i.e., physical education teachers, fitness professionals, and/or researchers) can utilize the evaluation rubric to improve existing videos and guide future video development.

Development and dissemination of instructor-led exercise videos aligns with the Comprehensive School Physical Activity Program (CSPAP) framework to promote youth physical activity outside of school hours, and within the home and community environments [33]. While the CSPAP framework is a comprehensive approach to promoting physical activity, primarily focusing on the school setting, it is vital to consider engaging families within the home and community environments. This study provides preliminary evidence that the InPACT at Home videos are of high quality. If future research confirms our findings using a more rigorous study design, then developing a collection of instructor-led exercise videos that induce low cognitive load may help to enhance youth physical activity participation in the home environment, which is the most underutilized (and under-researched) component of the CSPAP.

## Figures and Tables

**Table 1:** InPACT at Home exercise video evaluation rubric.

	Component	Implementation Level		
		Absent (0)	Developing (1)	Proficient (2)
**Technical**	**Visual quality**	The video is blurry; subject(s) isn’t framed well; unsteady camera work; etc.	The video only needs minor improvements to be high quality.	The visual aspects of the video are clear, framed well, steady, and appealing.
	**Audio quality**	The music is too loud and overpowers the narration; the trainer cannot be heard; the audio is spotty; etc.	The audio only needs minor improvements to be high quality.	The audio is well-balanced and clearly heard.
	**Matching modality**	The audio is out of sync with mouth movements; the audio is inappropriate for the visual aspects; etc.	The audio-visual components only need minor improvements to be high quality.	The audio and visual information are well coordinated to convey new information.
	**Signaling**	There are no text or symbols reinforcing information; the text or symbols are unclear; the text or symbols are inappropriate; etc.	The text or symbols only need minor improvements to be high quality.	Text or symbols are appropriately and clearly used to highlight important information.
**Content**	**Instructional objective**	There is no instructional objective.	The instructional objective is present but not explicit.	The instructional objective is explicit and clear.
	**Met objective**	The video did not address the instructional objective at all.	The video somewhat addressed the instructional objective.	The video fully met the instructional objective.
	**Call-to-action**	There are no recommended next steps for the learner to take after watching the video.	There is an implicit call to continued action for the learner.	There is an explicit call to continued action for the learner.
	**Bias**	There are intentionally biased statements or actions within the video.	There are unintentionally biased statements or actions within the video.	The content is presented without bias (e.g., gender, racial).
**Instructional**	**Learner engagement**	The information is presented in a vacuum without the learner’s engagement at the forefront.	The instructional techniques occasionally focus on learner engagement.	The instructional techniques fully focus on learner engagement.
	**Content organization**	The content is presented in a disjointed way with no logical organization.	The organization of the content only needs minor improvements to be high quality.	The content is presented in an organized way.
	**Segmenting**	The information is not broken down into chunks of information for easier learning.	There is some chunking but the video could benefit from more.	Short sequences of information are used to allow learners to engage.
	**Weeding**	There is an overabundance of personal or extraneous information that does not help build relationships or contribute to the learning goal.	There is some extraneous information that does not help build relationships or contribute to the learning goal.	All extraneous information that doesn’t contribute to the learning goal or help build relationships is eliminated.

## Data Availability

The data presented in this study are available on request from the corresponding author. The data are not publicly available due to privacy restrictions.
